# Gimme My Damn Data (and Let Patients Help!): The #GimmeMyDamnData Manifesto

**DOI:** 10.2196/17045

**Published:** 2019-11-22

**Authors:** Dave deBronkart, Gunther Eysenbach

**Affiliations:** 1 Society for Participatory Medicine Nashua, NH United States; 2 JMIR Publications Toronto, ON Canada

**Keywords:** data, participatory medicine, ehealth

## Abstract

Ten years ago, in 2009, “e-Patient Dave” deBronkart delivered an influential keynote speech at the Medicine 2.0 conference in Toronto, organized by the *Journal of Medical Internet Research’s* (JMIR’s) editor-in-chief Gunther Eysenbach, who themed the conference around the topics of participation, openness, collaboration, apomediation, and social networking to improve health care for the 21st century—with patient participation being a major component. Many see this as a defining event within the participatory medicine movement, perhaps the beginning of a social movement, similar to the women’s rights movement, with the title of Dave’s keynote “Gimme my damn data” becoming a rallying cry and hashtag for patients demanding more access to their electronic health records. On the occasion of the 20th anniversary of JMIR (and 10 years after the keynote), we are celebrating the impact of the keynote for the participatory medicine movement and #gimmemydamndata (also #GMDD) by publishing the transcript of these initial conversations as a manifesto of patients’ rights to access their data and their right to save their lives.

## Introduction by Gunther Eysenbach

Ladies and Gentlemen,

It is my great pleasure to welcome you to the second Medicine 2.0 Congress, also known as the World Congress on Social Networking and Web 2.0 Applications in Medicine, Healthcare, and Biomedical Research.

One of the most difficult tasks of being a chair of this meeting is, perhaps, that each year, I see it as one of my obligations to define or at least outline what we mean by Medicine 2.0 or Health 2.0, which seems to be getting increasingly difficult. Last time I checked, I found about three dozen different definitions of what people think Health 2.0 or Medicine 2.0 is. However, I hope that at the end of the two days, you come out of this meeting with sort of a warm, fuzzy feeling and you have a feel for what it is when we talk about Medicine 2.0. As a side note, I should maybe stress that I see the terms Health 2.0 and Medicine 2.0 as being largely interchangeable.

### Health Care Needs to Change

Despite this problem of lacking a clear definition, I think what brought us all together here—the kind of overarching theme of Medicine 2.0—is the recognition that health care needs change. No matter what country we are coming from—and by the way, there are people from 23 different countries here in this room—no matter what health care benefits we enjoy, no matter what health care system we are “enjoying” (or suffering from), no matter how we fund health care and what percentage of gross domestic product we invest in health care, what we all have in common as patients, consumers, and health care professionals is that we are becoming increasingly frustrated with the way health care is delivered, frustrated and impatient with medicine and health care, which often uses antiquated processes and technologies. Maybe one reason for this is that we are all part of the Google generation, and we are used to getting data, information, and answers at the click of a button, which seems to work in almost every other industry—except health care. Of course, there are reasons for that: privacy first and foremost. However, it is incredibly frustrating for us as consumers, patients, and health care professionals, especially for those of us who know that the barriers are not of a technological nature. There are so many things, which, from a technological point of view, are feasible. However, the barriers are mostly of an organizational nature, cultural nature, or political nature, and there is an increasing gap between what is technologically possible and what the status quo is. So, one possible way of looking at or defining Medicine 2.0 is to see it as “next-generation medicine”: what medicine and health care could be and what it should be, and hopefully, what it will be in the near future—if we all work together, if we are smart, if we reengineer health care using technology and participatory approaches, and if we reengineer health care using Web 2.0 values and approaches.

### Participation and Other Medicine 2.0 Values

Now, what do I mean by “Medicine 2.0 values”? I wrote a paper about Medicine 2.0 [1], where I outlined what, in my mind, the five characteristics of Medicine 2.0 or Health 2.0 are: (1) participation, (2) openness, (3) collaboration, (4) apomediation, and (5) social networking.

**Figure 1:**
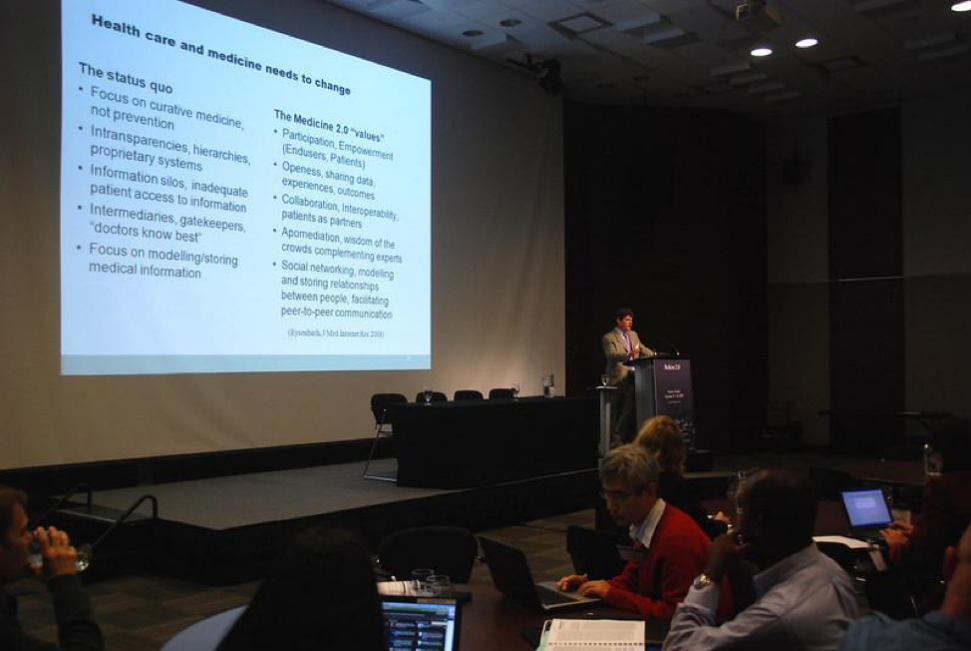
Current health care problems vs Medicine 2.0 (Eysenbach).

I think these five values or characteristics of Medicine 2.0 are the perfect remedies for what health care and medicine are suffering from in many countries, especially industrialized countries, worldwide (Figure 1).

These problems include, for example, a focus on curative medicine; insufficient incentives for prevention; and insufficient incentives to keep people out of the health care system, keep them healthy, and keep them out of hospitals. To keep people healthy, we need participation, specifically end-user participation. We need the participation, involvement, and engagement of consumers and patients, and that’s one of the values of Medicine 2.0: *participation*.

Health care around the world suffers in many cases from intransparencies and hierarchies, and if you look at computer systems, they are mostly proprietary systems, closed systems. Again, the Medicine 2.0 value of *openness*, sharing data, sharing experiences, and sharing outcomes provides a possible remedy to that. Health care is often characterized by information silos—inadequate patient access to information—and again, regarding the Medicine 2.0 value of *collaboration*, interoperability—the idea of patients as partners—provides an antidote to that.

In health care, we often deal with intermediaries, information brokers, and gatekeepers. On the other side of the equation is the Medicine 2.0 idea of wisdom of the crowds bypassing intermediaries, gatekeepers, and experts—not replacing them, but complementing them with wisdom of the crowds.

Lastly, social networking. Especially as medical informaticians, it is funny that we have focused our efforts on modeling medical information, and not on modeling and storing *relationships* between people. That is what social networking is all about.

In social networking, what we are trying to do is storing and modeling relationships between people, and that adds an entirely new dimension to what we deem as health care information. Social networks are fascinating because they also allow us to enable and facilitate collaboration between people. For example, they allow for collaborative filtering processes, enabling identification of relevant and trustworthy information based on what peers are doing, and for reputation and trust management. It allows viral dissemination of information and apps, which is a possibility that especially excites public health professionals, and you are going to hear some examples of that at this conference, for example, the use of Facebook to disseminate information. It is a potentially powerful tool to engage end users and patients in health behavior change apps and similar apps, because the common problem many of these fantastic Web-based behavior change tools suffer from is what I called the “law of attrition,” of people starting with a high degree of enthusiasm using these kinds of systems, but then they drop out. They discontinue the use of these kinds of systems. I think that if you add virtual communities or social networking, if you add some sort of peer pressure and social support to people to return to the website—not only peer pressure but some additional incentives, for example—they find their friends or peers there, that is a very powerful tool to engage patients.

So, some of the questions this conference hopes to answer or at least to raise are as follows:

- What are the implications of all these technologies for health and health care policy?
- How do the generic Web 2.0 concepts and technologies translate into health apps?
- What are the specific requirements for health-related or medical social networking apps?
- What are the research questions and issues and what are the methods to answer these questions?
- What are the determinants of success or failure?
- Is the “hype” promoted by private enterprises and venture capitalists supported by evidence and what can we expect for the future?

### Introducing “E-patient Dave”

It is now my great pleasure to introduce our keynote speaker, e-Patient (electronic patient) Dave deBronkart.

About 32 months ago, Dave was diagnosed with Stage 4, Grade 4 kidney cancer. It was bad, with metastasis to the lungs, several bones, and tongue. He read that his immediate survival time was 24 weeks. Using the internet in every way he could, he learned everything he could about his treatment options, joined an expert patient group on ACOR (Association of Cancer Online Resources), built a social network, and shared online medical records with anyone who could help. He beat the odds.

Having faced death, 2 years ago, he asked himself what he would do with his free replay in life and became a blogger about health care. Then, his doctor, Danny Sands, invited him to join the e-Patient movement, where he has become one of the most outspoken advocates for patient empowerment and patient engagement. As some of us who follow his blog know, he is a data guy. This March, when he clicked on the button to move his data from his hospital to Google Health, the mess that resulted was front-page news in the Boston Globe. He first blogged about it, and then, the media picked it up. That was around the time when I invited him to be a keynote speaker at Medicine 2.0.

His insightful blog post about the problems in interoperability and the publicity that followed led to him being invited to a lot of policy meetings in Washington. We are proud that we are the first conference where Dave is a keynote speaker, and I think it is not going to be the last one. Last month, he launched a consulting practice and just this week, he is featured with a full-page photo and a cover story entitled, “The Patient of the Future,” in Health Leaders magazine 

**Figure 2:**
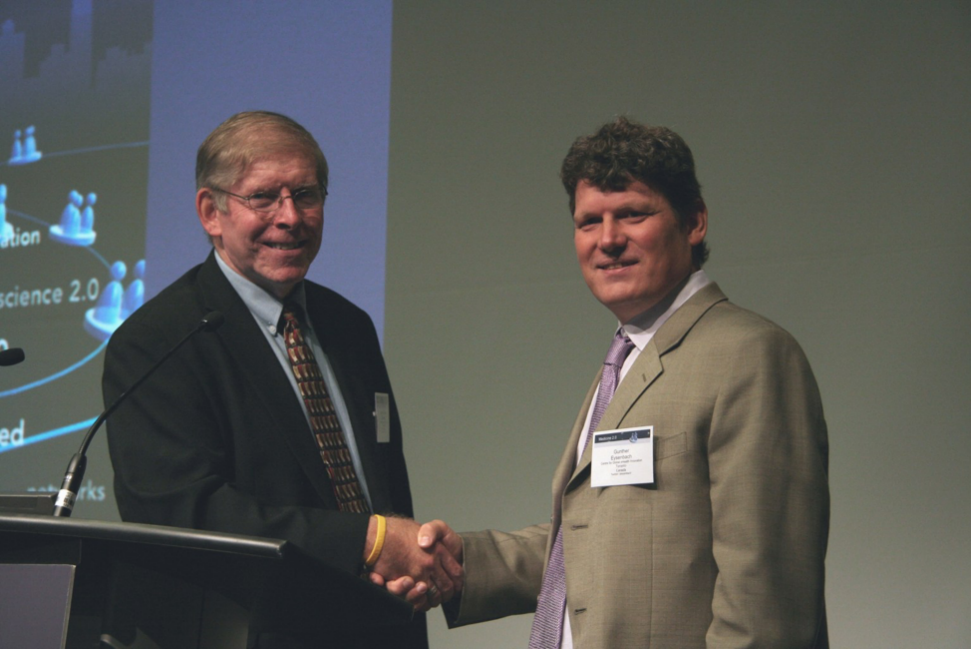
E-patient Dave deBronkart and Gunther Eysenbach (Founder and Chair) at Medicine 2.0 in Toronto in 2009.

Ladies and gentlemen, please join me in welcoming the “Patient of the Future”: e-Patient Dave (Figure 2).

## E-patient Dave deBronkart

### Introduction

What a thrill! Thank you so much! Gunther (Eysenbach), thank you for your vision. People who do not know the health care world are stunned when I tell them that a patient is giving the opening address at a conference like this. For those of you who do not know as much of Gunther’s history, he is one of the original visionaries who did the initial bit of research that, in a sense, opened up the whole world of participatory medicine: A number of years ago, people were concerned about the question of the danger of going out and googling, because we all know there is garbage out there. He researched it (among other things), and for years, tried to find a single case of death by googling, and he did not find it. He published that result. He even added a bounty after a few years of failure. That is a seminal moment, a vital piece of information in Tom Fergusons’ e-Patient paper, that I will talk about briefly later.

**Figure 3:**
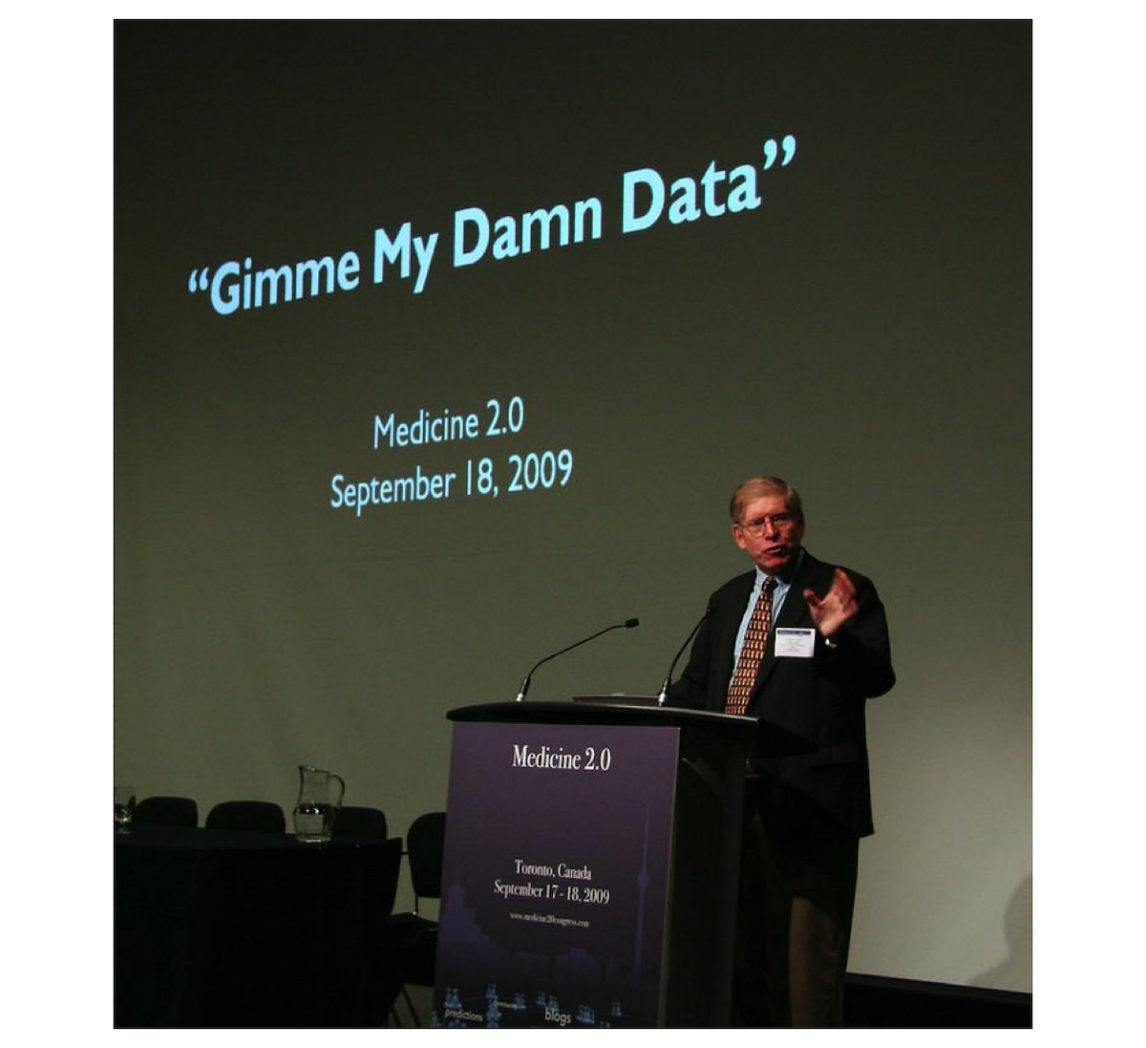
“E-patient Dave” deBronkart’s Opening Keynote at the Medicine 2.0 conference. [Editorial Note: The slide shows the wrong date; the actual date was September 17, 2009]

So, the title of this talk is indeed, “Gimme My Damn Data!” (Figure 3). The subtitle was going to be “Because You Can’t Be Trusted With It.” However, I toned it down a little because this is being recorded and broadcasted.

Talking about participatory medicine, the nice-looking guy on this slide, somebody thought that was clip art. No, that’s Tom Ferguson, the guy who founded the e-Patient movement and became an e-Patient himself in his later years. His movement has now become *The Society for Participatory Medicine*, which has just this year launched ParticipatoryMedicine.org. We are not really active on Twitter yet, but the address will be @S4PM. Aren’t you glad we did not spell out @participatorymedicine? I just hate long Twitter names.

### Foundation Principles

Here are some foundation principles:

1. Patient is not a third-person word. Your time will come
2. The right of a desperate person to save themselves
3. The right to know what your options are
4. The right to pick up your data and pursue an option elsewhere

I take this very seriously. Gunther told you a little bit about my story. However, my one story does not change the world. As I have become involved in this, I found myself, as Gunther said, asking myself, “What am I going to do with this free replay in life?” I found this calling to health care and as I started studying it. I have only been at this for a year and a half; the first session I went to was a small high-tech conference outside Boston. When my turn came to talk I said, “The thing I want us to shift here is that patient is not a third-person word, alright? We talk about patients as if it’s somebody that’s not here in the room right now. Well, trust me your time will come, alright?” and I went on at some length about that.

The next thing that came up: I went to a privacy meeting in Washington discussing several issues: the HIPAA (Health Insurance Portability and Accountability Act), data privacy, and others. We have so many obstructive regulations in place that it interferes with the right of a person to try to save themselves. Privacy and data security take on a whole different meaning when your life is at stake. I mean, look at it this way: We take our pants off for the doctor—why would we not share information to save our lives?

Then, “the right to know what your options are”: This is important with social networking (which helps to find these alternative options).

Ultimately, when necessary, the right to pick up your data and pursue an option elsewhere: “You know what? This isn’t working here. I want to go to this other hospital. Give me my data.”

### My Story and the Realities That We Deal With

Technological innovation can vastly alter things—iPods, cell phones, computers. However, health care is, in many ways, far behind other industries, and yet, the good news is it is not rocket science. The tools are available. Technologies are available. We just need to start doing this. Honestly, I am becoming impatient with the slow rate of change.

**Figure 4:**
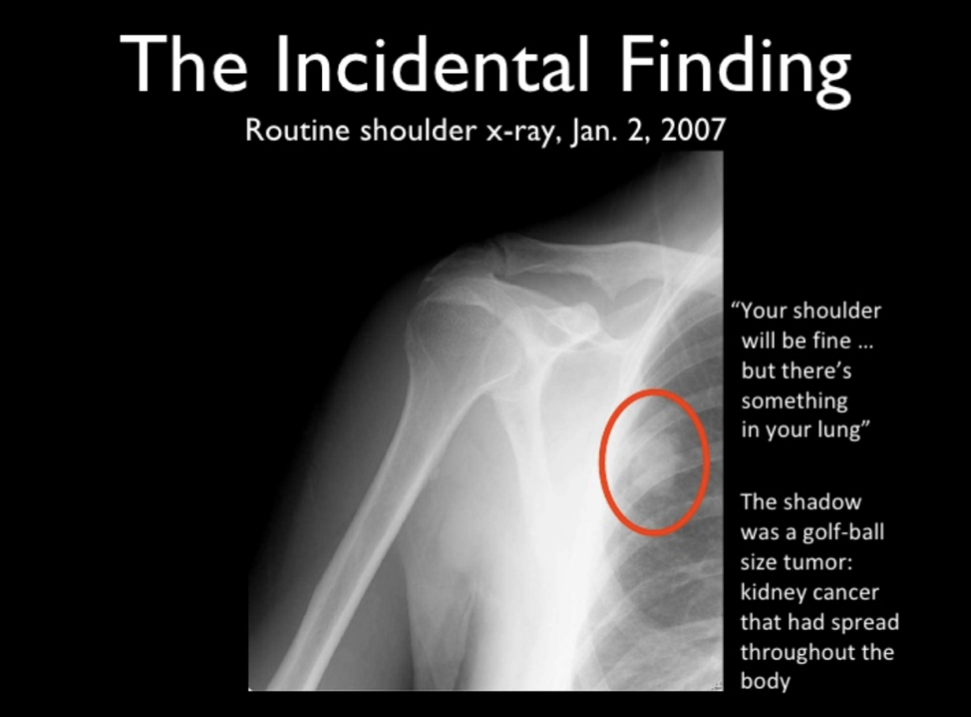
Dave’s x-Ray.

Here is a quick review of my story:

I had a sore shoulder at the time of my physical 3 years ago, and I got a shoulder x-ray. Now, those of you who know how to look at x-rays will see that there is something there, that shadow should not be there (Figure 4). Totally by coincidence, the shoulder x-ray picked up a golf ball–size lesion in my lung that happened to be near the shoulder. That shoulder x-ray saved my life because I did not have any physical symptoms until 6 weeks later and by then, it would have been too late.

So, that was ultimately found to be kidney cancer that had spread throughout my body. I was near the end. So, I researched (I have always been a Googler) as I like to say, I googled my butt off. This happens to be a graphic from the website of the treatment that I got, which was high-dosage interleukin.

Indeed, in every one of those spots, I had a tumor. I had one in my skull, numerous throughout my lungs. The one in the femur was so big that eventually, the leg broke. I now have a nice steel leg. That is a pretty big metastasis when it breaks your leg. Here are the others. There was one in my ulna. When the leg was in the process of breaking, I could not use a regular walker because it put too much stress on the ulna, which also had metastases in it. Finally, one in my thigh muscle and in my tongue. That is gross. I am glad that happened just before the treatment started because it pretty quickly would have fallen off.

It is sobering. I mean, I have always been somebody who has been able to find ways around things. Whether it is computer software or car research, I do not do well with being told, “You can’t do that.” However, every place I looked, I read things like, “The prognosis is poor. Almost all patients with Stage 4 Renal cell cancer are incurable.” I remember the night that the biopsy finally confirmed the diagnosis, and I read “24 weeks survival time.” Now, this was in January. I remember waking up at 1 AM one morning and thinking, “I might not see Christmas. I probably will not see my daughter’s wedding.” I actually had the image of seeing my mother’s face at my funeral, and I faced the task of sitting with my daughter and her boyfriend and giving them the news and telling them that they had better not get married prematurely just so they could do it while I was alive.

**Figure 5:**
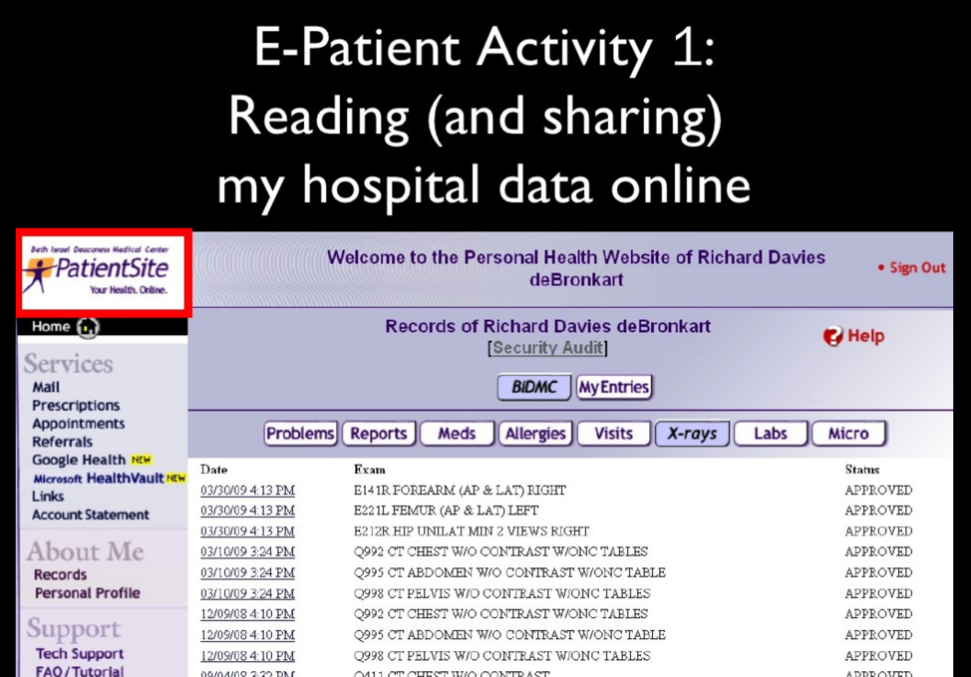
Dave’s personal health website at Beth Israel Deaconess Medical Center.

So, you are left with several questions: “What are my options?” “What can I do?” “How can I get myself into gear?” So, you get engaged. You do everything you can. First thing I did was go to PatientSite, my hospital’s personal health record system (Figure 5). It is ugly and really needs a makeover. However, you know what? I could look at my data, and I could give my password to other people so that they could look at it and give me advice and feedback as well.

### Patient Communities

**Figure 6:**
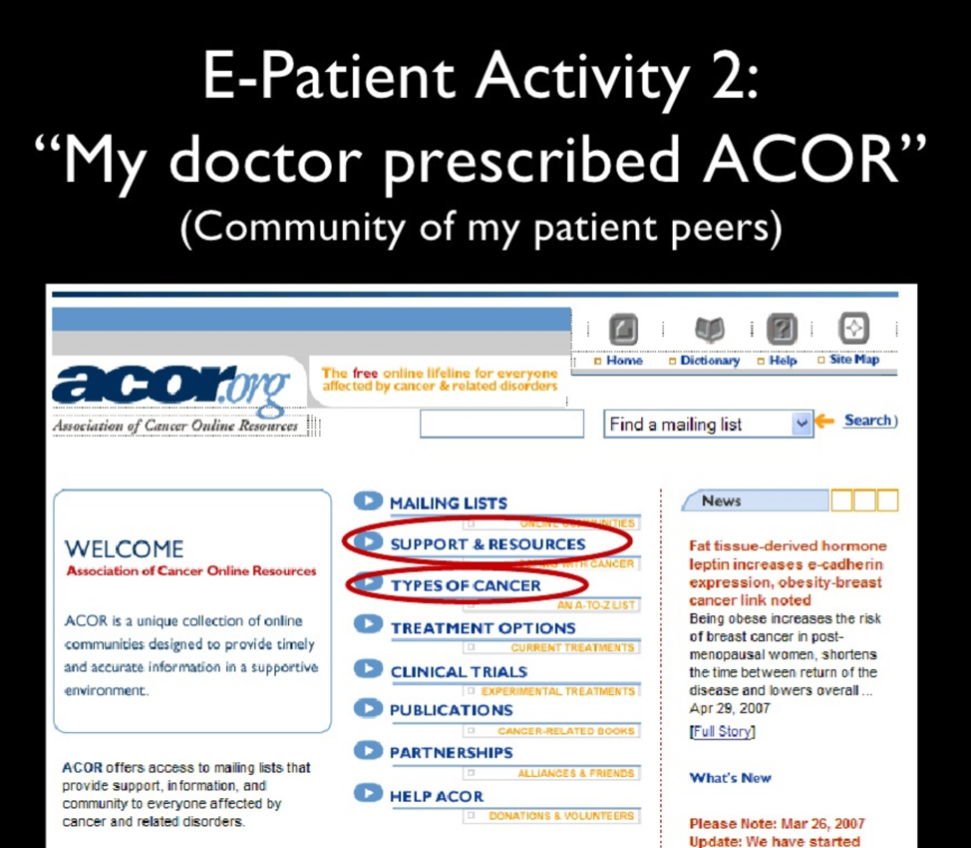
The Association of Cancer Online Resources’ cancer patient communities.

 The second thing I did, I joined ACOR (Association of Cancer Online Resources). I love this phrase: “My doctor prescribed ACOR” (Figure 6). My doctor handed me a prescription slip with “ACOR.org” written on it [Editor’s note: This is now SmartPatients.com], and I found there was more useful, action-worthy information from other patients than I found on any encyclopedia-style website. Encyclopedia-style websites could give me peer-reviewed information that was 10 years out of date and still could not tell me anything about what the treatments were really going to be like.

Patient communities are responsive. People discuss what to do, and patients know what patients want to talk about. Consider this, we talk about referral delays reaching a doctor. Well, I got responses in 4 minutes and 11 minutes to my first questions in the patient community.

This is an appeal I always toss out: Whatever we do in health care spending, we should just devote 1% of it to funding patient communities, to let patients do what they see as necessary. For those of us who think about how hard it is to get physicians to adopt medical record systems, consider this: Down, at the bottom, is an audience who you do not have to motivate for adoption. These people are already motivated and engaged.

**Figure 7:**
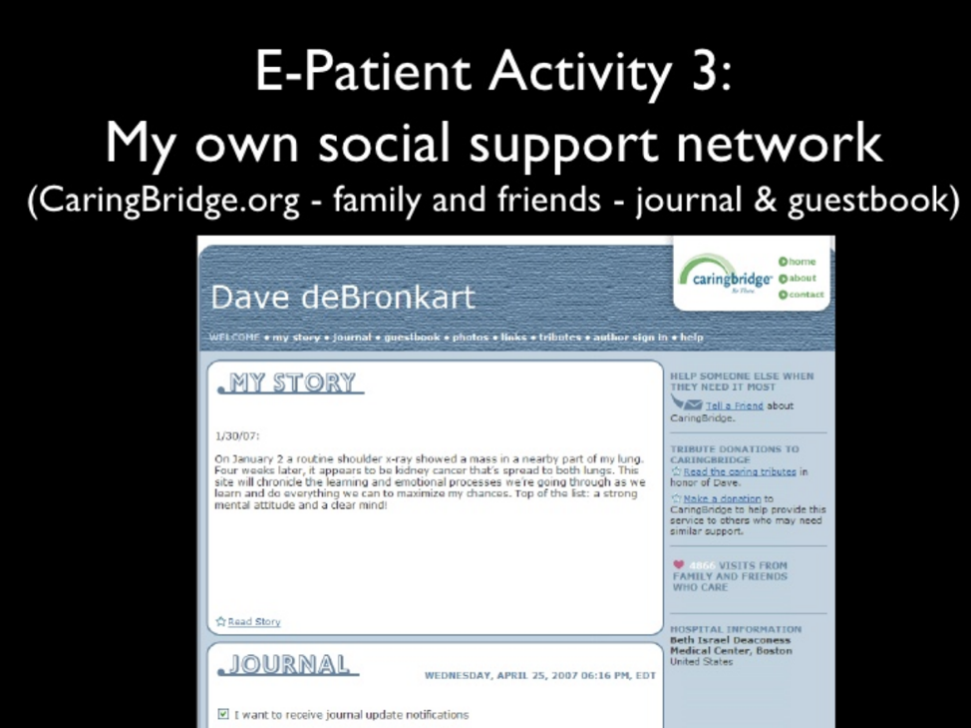
Dave’s own social support network.

Finally, my own social support network (Figure 7).

### Participatory Medicine

Now, long story short, because I have other things I want to get onto, the treatment worked. Surprise, surprise! I made sure I got copies of all my scan images. There was one of the lesions on the left side before. If you consider the size of my midsection, that is was a pretty good-size tumor. That little white dot is what was left of it 50 weeks later. The tumors have continued to shrink, and the ones that remain have been stable and are considered to be dead. So basically, I won. So, what do I do now?

In January of 2008, as Gunther said, my physician Danny Sands invited me to join this group he belongs to, “Equipped, Engaged, Empowered, and Enabled: E-patients - A Different Kind of Beast.” There is a white paper by Tom Ferguson, that, if you have not read, this is the paper that mentions Gunther’s contribution as well. It is at the top of the e-Patients.net website. One key point was, and this was a mind pop for me, that Donald Lindberg, the head of the National Library of Medicine said, “If I read two journal articles every night, at the end of a year I’d be 400 years behind.” What was a mind pop about this for me was, “Okay, I can help.” It is not a case of the doctors knowing everything they need to know. I can help.

Then, there is the other important point: the lethal lag time.

The publication delay between the time information comes into reality and the time it takes to make its way through the publication pipeline is 2-5 years. In fact, from what I have heard, the time from when an idea is conceived until the research is conducted and the results are out there can be 17 years. During that time, people can die due to a lack of information that exists. The internet can solve this. Tom Ferguson had this early vision of how accessed information would turn health care on its head. These slides are on e-Patients.net too. The idea here, with industrial-age medicine before the internet, was, if you look at the bottom, that the ability to create value, like in the 1800s, accrued to people who owned a factory. That was the means of production. However, the information-age medicine turned health care on its head and now, what we have are individual self-care friends and family. The latest Pew Internet research data says that people turn first to friends and family and then to self-help networks like ACOR and then to professionals as facilitators. I will come back to this point of professionals as facilitators.

The key is that the internet gives us access to each other and to information that, in the past, we could not get at—and that changes everything when you are desperate. That is participatory medicine. Tom Ferguson saw this within a year of birth of the Mozilla browser. That was an amazing insight. He published those slides in January 1995.

Regarding this point about participatory medicine, I wrote a post in December about Stanley Feld, a physician who had written a post called, “Physicians Are Coaches and Patients Are Players.” I will not spend time on it here, but think about this. In reality, physicians advise us on what to do, but we do not always do it. For example, I take my medication, but exercise and eat better? Well, not so much. What you start to see, when you give up the viewpoint, is that although they have all the knowledge and power, “a lot is up to me as to whether I do what they say.” Things start to shift. More about that later.

### Decentralization Follows Centralization

I work in high-tech software marketing. I have been involved in high-tech industries around Boston for my whole career. This spring, I met Clayton Christensen, the author of *The Innovator’s Prescription*, and all of the innovator’s dilemma books. He has studied this for years and has come up with a really world-changing perspective on how new technology changes an industry. His health care book was published in February. There are a lot of people who disagree with it. I do not assert that everything he says is the solution, but I want you to think about something that I know, from my personal experience, is really accurate.

In the bottom left is an archaic device called a slide rule. I used to own one, and one of Christensen’s points is that the centralization of a skill or a technology is followed by decentralization, and here is how that played out in computing:

We have here a picture of an old-fashioned computer room, the kind of thing where, when I was in college, we would take a stack of Hollerith cards to the computer room and hand it in and come back the next day to get our printouts: This involved highly specialized skills, expensive equipment, and specialized environment. As time went by, it got to the point where things were more understood and you could have a simpler, smaller computer in a less specialized area. So, you had departmental mini-computers. Then, it got to the point where we had desktop computers, and then, ultimately, laptops, and now, today, it is on our hip with a smartphone. These innovations depend on the processes becoming reliable, so you no longer need a computer department geek *[sic]* to run the machine, and as the data is mobile, it can move around. The decentralization that follows centralization is only beginning in health care, but it is beginning.

Here are three pictures of a family doctor, like we had when I was growing up, who would come to our house and in the center now, like a computer room, we have the massive general hospital. I met Christensen at a meeting at Beth Israel Deaconess in Boston. It was a promotional event they were doing to encourage people to fund research there. Christensen stood there and looked Paul Levy, the Chief Executive Officer, in the eye and said, “The general hospital is not a sustainable business model. It cannot survive without philanthropy and government subsidies.” That is a pretty challenging thing to think about. Why? I will get to that in a moment.

So, here you have, as with computer departments, all kinds of specialized equipment and the general hospital is set up to be able to do anything for anybody. What has happened now is that there are smaller regional medical centers that can do a lot of things that used to require going downtown or to the big centers. Now, there is more technology in the physician’s offices, and finally more things are being done at home with connected health equipment and self-monitoring, and this is only the beginning.

Here is a diagram that Christensen used for this—a metaphor of a general hospital. This is a diagram of a hypothetical factory, a machine tool department that can do just about anything for anyone. So, with all these work stations, a particular product might start at one station and wander all over creation. This is the path taken by product A through this shop. This is what it is like when a patient goes through a general hospital with a complex condition. Here is a different product that goes through a completely different path.

Regarding this, I met with Jason Wong, the co-author of the book and said, “Do I understand this correctly? Alright, let’s say instead of that being an assembly line, let’s say this is someone’s life. So over on the left, you have ‘Life starts here’ instead of ‘Product A starts here’ and ‘this is the path taken of life.’” This starburst here, this is the moment of my diagnosis, the moment of awareness. Everything in Christensen’s and Wong’s book is about the delivery of care after the diagnosis when it is recognized that something needs to be done. What is not mentioned there is that today with new technology and social networks, values being created outside the hospital walls and outside the health care delivery systems, what if this awareness happened earlier in the process? What is the impact of having the information earlier? Well, in my particular case, as a kind of a trivial example, I got diagnosed 6 weeks earlier, so I am alive. That is an impact that I care about. On the other hand, there are other ways that we could do better and be more aware of our well-being. Making a difference back at this point requires two things:

- Data: The evidence of what is happening
- Knowledge: The meaning of the data and what to do about it

That is the meaning of an engaged, empowered patient with participating providers. This is how it goes differently. You are more aware if you have the information and the knowledge of what to do about it.

### Google Earth for My Body

Here is another example of this—and Gunther talked about technologies that are out there—and we are starting to use them to match them up in ways that have not been anticipated.

One of the tumors in my kidney was encroaching on the psoas muscle. Now, I had no idea what a psoas muscle was. I went to visiblebody.com, which is this amazing, free, interactive, 3D website. I cannot believe they can do this for free: You can turn on the skeletal system, the nervous system, and so on. You can click on things to remove them, and I was able to create this image of the relationship between my kidney and the psoas muscle and then rotate it in 3D. Look through. I thought, “What the heck!” We have Google Earth where you can fly to Duluth and go to an individual building; why not Google Earth for my body? This is a generic body, but it is not hard to imagine that we take my scan image, which is currently a crummy, black-and-white, low-resolution thing and mash that up with this, so that we can actually look at my body in 3D. I can tell you from a visceral level, I have a different relationship to what is going on inside me, given this awareness of where the pieces are.

This is the article “Patient of the Future” from this week’s *Health Leaders* magazine that Gunther mentioned. You can see this online. I’ve got it here because I just love the vision that the woman, Gienna Shaw, articulated. She was new to this whole subject, but she completely got it:

Patient enters the waiting room and is greeted by her personal navigator who hands her a tablet size computer. In his office, the physician is reading an email from a patient who has forwarded an interesting study.

There was a great moment almost two years ago where I was waiting for a CAT scan follow-up visit in the doctor’s office. While I was waiting, I was on the computer doing some stuff, the oncologist and the nurse practitioners came in, and a few minutes later, my wife cracked up. She was sitting there. She said, “What’s wrong with this picture?” because I was sitting there pointing something out on the computer while the oncologist sat on the examining table and the nurse practitioners were writing down the URL. It is a new world, people, completely new world.

### Where Are We Today? The Google Health Disaster

So, that is the vision. Where are we today? I went to move my data into an online personal health record. So, I punched the button to move my data into Google Health, and I got this craziness. I got this false medication warning saying that my blood pressure medication conflicted with low potassium in my blood. Well, low potassium in my blood was true when I was in the hospital being treated for the cancer. It was not accurate. Plus, there was a whole bunch of things: In my condition list, on the right side, they listed everything I had ever had with no dates attached to it, which was crazy. We looked into it, and it turns out that they had transmitted billing codes instead of clinical data, and, from an IT (information technology) perspective, it is just goofy to pick up one type of data just because it happens to be available and use it even though it is not appropriately modeled for what you are going to do with it.

Here is the thing: It is a long story. You can read the 3300-word blog post about it on e-Patients.net if you want. I have to say that both the hospital and Google Health responded in an exemplary way. They completely disclosed everything. Well, once it hit the front page of the Boston Globe, they responded in an exemplary way. However, I got a complete Excel spreadsheet of every billing record that the hospital had for me, and I went through and added notes on the left. My physician went through and added notes on the right. We had some crazy things like the top note on the right points to an item 424.2 “non-rheumatoid tricuspid valve disease.” Well, for one thing, I never had that, and for another, that was noted during a visit where I was getting an infusion to treat my bone metastases. It is not normal for a cardiac condition to be diagnosed during an orthopedic visit.

Down at the bottom, bone and cartilage disease. That was during the visit where I went in when the tumor had erupted from my tongue. How that billing code got in there, nobody knows. These are not data that are well managed, and this is at a hospital, by the way, that is well known for being one of the leading hospitals in handling IT.

Another example is the top right “Aortic Aneurysm.” Well, it turns out that on one report, a radiology report showed a 1/4-inch enlargement at the base of the aorta, and strictly speaking, that is an aneurysm. Do you know what the trick is? Upcoding. You can bill more if an aneurysm is involved than if there is a slight swelling involved. When I learned about upcoding, I immediately thought about my supermarket, wherein the deli section, they have regular bologna and then they have, next to it, something that looks awfully similar, labeled “Tasty, delicious bologna” and it is priced higher. So, here we have tasty delicious bologna applied to our health care cost.

Then, there are other things like volvulus of the intestine. This is a life-threatening kink in the intestine, which is fatal if not fixed in a couple of days. I have never had it. How it got in, I do not know. How much of the excess health care cost in the United States is due to things like this, and nobody can track it down. So, physician errors, clerical errors, and upcoding.

Here is the thing, HIT (health IT) just needs to follow normal IT best practices. Find the right data vocabulary. Use good reliability practices and test them with real-world data before going live. It is not rocket science; it is available to us.

Here is another risk that nobody talks about. In the United States, there is an organization called the MIB (Medical Information Bank): It is an insurance industry association where insurance companies share what things you have had billing codes submitted for, so that you cannot hide conditions from them such that they might be harmed by your dishonesty. However, this video from Consumer Reports is about a woman from the Katrina area in Mississippi who lost her 401K savings and everything else. She has asthma. Her insurance company would not cover her treatments, and it turns out the root cause was that her physician had accidentally miscoded something and it took her forever to find that out.

The MIB is a private insurance industry data bank. I got in touch with them. They are totally opaque. You cannot find out stuff in there. Because they are similar to a credit bureau, you are allowed to get a copy of your records, but as I wrote on the blog, and I would not go into it here, but good luck making sense out of it. Their lawyers started writing to me and made clear that they do not consider themselves liable for any damage they cause. So, the lesson? You better check for errors. If you are an American, check into the MIB.

Here is the question, what is in your wallet medically? Do you personally know everything that is in your medical record? HealthDataRights.org published the declaration of health data rights this summer. You can go sign it, endorse it if you want to. At Health Camp Toronto, yesterday, we had a terrific discussion initiated by Jen McCabe who will be on later this morning saying that this is not tough enough, that one thing that is not stated here is the fundamental thing: This is my data. It is my property. I mean, whose data is it? So, we are actually thinking about revising that. My endorsement said, “Look, these rights are as inalienable as the right to life itself. Whose life depends on its accuracy? Whose data is it anyway?” My physician in his endorsement said, “How can patients participate if they can’t see the same data?”

To wrap up, Clay Shirky is a real internet visionary. For instance, if you are familiar with the concept of the long tail, which really alters our thinking about finding things on the internet, he is one of the developers of that idea. On the subject of data sharing, he said, “Giving patients access to their medical records will naturally improve the quality of what’s in there.” I love the way he said it. You clean up when you know company is coming, when you know somebody is going to be looking at the data. He also pointed out the opposite model—clean then share never works out because it turns into, “Well, we can’t share it yet because we haven’t cleaned it yet,” and in the meanwhile, lives are at stake.

Finally, Gunther talked about Medicine 1.0 and Medicine 2.0. We had Web 1.0, which was publish-only. Web 2.0 is read-write. We can put things out on the Web. We can share information with each other. No less an authority than Tim Berners-Lee, considered the founder of the Web, gave a talk at TED Talks this spring where he talked about what is next for the internet, and it’s beyond anything I had realized. It is not just aggrading similar pieces of published data. He is talking about intelligent agent software that can go out and derive new information, create new knowledge. However, it cannot do it if it is looking at other people’s interpretations. It has to be looking at the raw data. So, his “TED Talk” is a great video. It ends with him actually getting people to start chanting, “Raw data now! Raw data now!” We, I think, ought to be thinking along similar lines.

Takeaways: It is my data. It is your data. Innovation depends on good-quality data and reliable workflows. Let us help.

### Let Us Get Involved—Give Us Our Data!

I got to the point this year, where I said I cannot keep doing the day job and do this at night. So, this is my disclosure of financial interest. I have a financial interest in keeping myself funded so that I can do this. I have gone into business for myself.

To wrap it all up, here are 2.8 years in pictures. This is me 3 days before that diagnostic x-ray. I did not realize it at the time, but I was not looking too good. Then, by Halloween, in October, at the company Halloween party, this was me kind of grinning at the reaper. I did not lose my hair due to chemotherapy. Kidney cancer does not use chemotherapy. However, my hair became an absolute mess, and I just said screw it and chopped it all off. So, that is me very happy 10 months later. This is me with my mom, not at my funeral, but at my daughter’s wedding in May. It was a great thing. It was wonderful. Here is a real kicker for you. This is me last Sunday with my bone surgeon, because somehow, something has happened to me and I have decided to get interested in my health, and I rode a bike in a cancer fundraiser. I have not ridden a bike since I was 20 years old. However, this June, I said to my wife, “You know what? I want a bike.” We went and got a yard sale bike, and it turned out that I loved it and I was good at it. Just to show that this health care stuff is really serious, I lifted up my shorts, got out a magic marker, and wrote a note to my bone surgeon, “cut here,” to avoid any chance of any wrong side errors.

That is it. Here is something to think about. Could it be that engaged patients might take better care of themselves? Once you really get that all the knowledge that is not out there, that I have something to do with it, that is my coach. I am the one on the court playing the game. So, let us get involved. Give us our data!

Thank you.

## Q&A With Introduction by Gunther Eysenbach

Thanks very much, Dave. You almost got people chanting, “Give us our data!”

We have about 25 minutes for discussion and questions. In general, we try to leave ample time for discussion at this meeting because we want this to be an interactive meeting. There are two microphones here in the auditorium coming around. First question:

### Audience Member #1: “It is a Social Question”

Dave, I mean, you are absolutely correct. I applaud you on a really excellent presentation, and congratulations on beating your cancer. You are a very strong individual and someone to be looked up to.

I think that you have hit upon one of the most essential issues in health care today, and I can tell you that as a physician, I remember treating patients, and I have treated patients, but I remember one, in particular, that came to me with lesions in the brain and needed a tumor resected. I had actually already received this information and someone suggested just watching it. We removed the tumor and found out it was a melanoma. After surgery, she came back and we were talking. The question came up, “Have you ever been diagnosed with melanoma before?” The answer was, “No. Never had a problem.” She left the office, I was seeing the next patient, and she came back in. She popped her head in the window and said, “You know, I did have a skin biopsy about two years ago. I assumed it was normal. I never heard anything about it.” I actually knew the dermatologist. He was in the same building that I was working in. I called him, excellent reputation, excellent dermatologist. I mean, renowned in the area, and I asked him to look up the biopsy results. He said, “Yeah sure, Luke, you know. I’ll be right back.” He came and there was a moment of silence. I heard him rustling papers. After a moment of silence, he says, “I don’t know how this happened, but the result was in the chart. It was melanoma. There’s no signature. I sign and date every paper I’ve seen. Someone in my office popped it into the paper chart without my having a chance to review it.” The patient was upset, of course. However, she accepted it as human error, and she passed on about 7 months later.

Since then and with other episodes, I see this happen over and over again. The issue is essentially that: How do you get the data to the patient? It is really not even a technology question. *It is a social question*. Just getting your own data, even as a physician, out of a hospital is almost impossible. Sometimes, it takes days to get the data extracted. I have colleagues, friends in a project that I am working on, who basically have children with extended stays after birth with long records, and they cannot get the records to bring to the pediatrician, so the pediatrician can be aware. So, you hit right on it! How do we fix this? In other words, doctors will point to things like HIPAA and say, “Oh, I can’t fax these records to you because of HIPAA laws.” Total falsehood. It is not true. It is not just technology that we need. We need someone to say, you have a right to those records within 24 hours, and at this price, that should not be a state or federal issue; it should be an international issue. It is a human right, and I totally agree with you. How do you make that happen?

### Dave deBronkart

You touched on a lot of really powerful things there. How do you make it happen? Get started for heaven’s sake!

For another thing, I would recommend spreading the word about that Health Data Rights Declaration.

On the subject of that melanoma information going into that folder without being reviewed, you may have heard earlier this summer, there was a study, which announced that 1 in 14 lab results do not get communicated to the patient—7%! Now, imagine if, just take your head out of health care, and imagine if 7% of credit card transactions never ended up on a statement. If it is your credit card, you might be happy about this, but the merchant certainly would not. Here is the thing, at work, in my day job, where I still work part time, I manage the sales automation system called SalesForce.com. We can set up workflows that say, “after three days, send an email to somebody to remind them of this” or “when a new inquiry comes in, automatically send out a thank you email of the following form.” These tools exist, and you know how much it costs to get started a big, hairy, automated system? Well, nothing. You go to salesforce.com, you click “free trial,” and you start using it. That is cloud computing.

Not only that, the cloud can also securely back up your data. I was at a conference, the TEPR Conference in Palm Springs in February, and a high-level AMA (American Medical Association) official talked about the nightmare that happened when he installed his medical record system. Now, if somebody had tried to infiltrate a medical records conference with a horror story to defer adoption, this would have been a great way to do it. He lost all his data. The guy who manages his database could not get it back, and the following speaker said, “Don’t you know about cloud computing? We have all our data stored in Google Docs and SalesForce.com. They do the backups for us.” So, there are technologies out there.

Now, I will say, if you think back to when online banking first started, the early websites were ugly and hard to use, and there would be mistakes and issues. It took about 10 years to get it right and make it reliable. The clue is to get it started and certainly encourage each other to get off of the objection that it is too hard. It can be done.

### Audience Member #2: “How to Change the Culture So Physicians Work Together With Patients?”

Hi, I am from Italy. This is the first time I am participating in Medicine 2.0. It is a wonderful venue, and actually, your talk was really engaging. I was very glad to hear your story. I would like you to comment on how participating in the decisions of medicine and physicians means a cultural change, which is possibly the most difficult thing to do. Actually, what happens most of the time, Dave, in my country, is, for example, the community of patients stay together and talk together about the potential therapies and alternative therapies, and they are very far from the decisions of their physicians. At the same time, physicians do not believe that they are looking for the right things, and the gap is growing. You said one very important thing is that the physician is a coach and I am a player. Actually, I think this is the key, but this is very difficult. How can physicians work together with patients to find the right solution? This is, I think, the key to get into the right process of patient participation in medical decisions.

### Dave deBronkart: “Doctors Should Learn to Say ‘I Do Not Know’”

That is a great question, and I want to make clear that I know there are idiots out there. There are idiot patients, and there are crooked doctors. Big surprise! I think that is true in every category of life. The way my physician, Danny Sands, expresses it is, “Embrace knowledge asymmetry, the idea that there can be knowledge on both sides of the equation.”

In a nonmedical conference a year ago in San Francisco, somebody walked up to me, having heard that I had cancer, and said, “You know, chemo is a fraud. All you need is properly ionized water.” I was like, “Well, thank you very much.” So, although there are not known cases of death by googling, you can come up with some idiotic things.

Danny also mentioned at another conference that “in medical school, we are taught all kinds of things about how to have certain kinds of conversations with patients, but what we are not taught how to say is, ‘I don’t know. Let’s find out.’” That is something that he is really good at and I know that. I know that personally, from my personal interactions with him. Is that a sufficient answer? I think also, by the way, the Society for Participatory Medicine is going to be working on developing advice and principles and practices for how to do that. So, you know, if you go sign up, you can participate in those discussions.

### Audience Member #3: “How to Address the Lethal Lag Time”

My particular interest is in medical research and your statement about the lethal lag time: What do you think the solution is? Should we dump the traditional method of randomized controlled clinical trials in three years till publishing the results? Should we get rid of peer review or thinking radical ideas like that or just other methods to solve this electronically and reduce that lethal lag time?

### Dave deBronkart: “What I Want Is Access to Useful Information When You Are Desperate”

That is a really good question because I know there are limitations to the peer-review process. I wrote a post last year about evidence-based medicine, which I had just learned about. Please understand the world that I live in. The good news is, for me, medically, I am now at the point where I only have a 50% chance or probability or the cancer coming back. There is a 50% chance or probability that I will be back there someday. I am doing everything I can in the way of attitude and finding information. But the fact is, my oncologist says, “We have no more advice for you on what you can do.” So, here’s a question. When you’re at the fringes of medical knowledge, and the doctors have no answers, and your life is at stake, what do you do? Well, I’m not going to pitch out the whole peer-review process, where you have people who can say, “Wait a minute. This is a crock. This is poorly thought out and so on.” At the same time, I know there are serious limitations to that. The reporting of relative risk reduction rather than absolute numbers is something I have learned about. Now, the Lipitor, on which we spend US $25 billion a year, actually affects one patient in 200 that takes it, if you get down to the raw numbers.

So, that is just the discussion of the peer-review process that I think is a good thing and needs to be understood for what it is and is not. On the other hand, what about when your life is at stake and the information has not been published yet? What I would like to see—Judy, you did something like this, right? Judy Feder, an astounding e-Patient woman who went and found unpublished information for a test that led to a treatment that has produced really favorable results.

So, I think, what I would really love to see is a very open database of all the studies that have been attempted or are in process, so that people with their physicians can get at those and find out what is out there, because if you are out of peer-reviewed stuff, you might as well look at the stuff that is not vetted yet.

A really good example of [communication problems regarding lag issues] is the following: The Kidney Cancer Association did a patient day two years ago in Cambridge, which was really so old school, old guard. What they did is they look at what doctors talk to each other about and tried to present it in patient terms. When question time came, I stood up and, at first, thanked the physician, because he was the head of the program at Beth Israel that had saved my life. I wanted to make sure he knew I was not just going to be doctor bashing. Then, I said, “If I understand it correctly—and this is based on stuff I learned from my patient community—the numbers you just put up there for median survival time and everything, those are from the Kansas City Kidney Cancer Base, correct?” He said, “Yes.” I said, “If I understand correctly, all the data for that was collected ending in 1996, correct?” He said yes. “And at that time none of today’s treatments existed, correct?” He said, “That’s right.” I said, “So, those numbers have nothing to do with somebody’s expectations if they just got diagnosed today, right?” He said, “Well, that’s correct.” I said, “Well, I wish you would tell people that, you know, because you just scared half to death a guy at my table who’s newly diagnosed!”

There is this discipline of saying, “What we will talk about is the latest peer-reviewed randomly controlled trial...” which may not be the most useful information. So, what I want is access to useful information when you are desperate.

### Audience Member #4 (Judy Feder)

To your point, do you simply throw out the peer-reviewed research? No. However, I firmly believe that there is a place where evidence-based and experiential data have to meet and that we are smart enough and flexible enough, our brains are flexible enough, to figure out a way to negotiate that ground. I had a real epiphany a few months ago when I was telling somebody my story that I was being treated based on data that really was not “proven,” and they said, “Well, this raises all kinds of regulatory and you know, liability issues and all kinds of things. You know, how did this happen?” I said, “Well, I didn’t walk into my doctor and say, ‘Hey, I would like Herceptin.’” The doctor said, “Yippee! Let’s give you Herceptin.” My doctor said, “Really interesting information. Let’s see what the evidence says.” So, that is the process that needs to go on. I mean, it needs to get vetted from multiple standpoints, but I think we are caught in this black-and-white thing that either it is what the doctors say from on high or it is the patient’s report in their free-form discussions, and we are really trying to get to a medium.

### Dave deBronkart

Yeah, that is absolutely perfect.

### Audience Member #5 (Lisa)

Following up on what Judy was saying, one of the issues that you did not raise was that of the poor health literacy skills that far too many patients have, and I do not know what yours were to start with, but you developed wonderful literacy skills. However, for all the good e-Patients, there are also the many people who are looking for miracle cures and are very easily swayed by the far-too-abundant information that is out there that leads them in very negative paths, perhaps, not as Gunther showed, to death, but certainly to avoid appropriate medical care because they are following treatments. Working with Alzheimer patients, a lot of people follow advice, which is actually detrimental to their health, and certainly that is true with a lot of other diseases as well. However, it is also true around much simpler issues like weight loss, where people are looking for miracle cures and easy answers. So, I think that it is important to look at health literacy issues, and it is also important to look at the path that gets somebody to being an adept e-Patient.

### Dave deBronkart

You know, Lisa, that is a great point, and it really goes to one of the most foundational issues in all of this. Alan Green from drgreen.com, who is the President of The Society for Participatory Medicine, is also quite a history student, and he has this array of Thomas Jefferson quotes that talk about this. From the founding of this country, well, that country over there—the United States—something about if the problem is that the people do not know enough to govern responsibly, the solution is to educate them, and at some point, you have to give people, like “Okay, I’m taking my hand off the bicycle. You’re on your own now. You know, you’ve got to make your own choices.” For better or worse, that is the nature of democracy as well, and it has effects like the very weird things that are being said in the health care reform arguments in the United States now and so on.

It is a real issue, and it is why there is going to be a real need and real value in it. I know that is the work you do to help people achieve health literacy. Yes?

### Audience Member #6

Thank you. I want to thank you for a really inspiring talk. Just to this point of coaching, there is a website called QuestionsAretheAnswer.com that was just published on Medicine on the net that helps patients communicate and ask the right questions to their doctor. There is a much older one that I am sure everybody here knows about, called “Quack Watch,” that helps patients figure out what are the quack solutions out there.

I also wanted to ask about Tom Ferguson. I did not know he was ill. I met him in 1995 at a Leaders in Telemedicine conference, and he was an inspiring man.

### Dave deBronkart

Yes, well, he had multiple myeloma. He was an actual e-Patient himself, and amazingly through unconventional channels, he learned that thalidomide, which had caused all those birth defects in the 1960s, was really useful for his condition, and it prolonged his life for years. He died unexpectedly in 2006.

### Audience Member #6

He did. Oh, I am sorry to hear that.

### Dave deBronkart

In an interesting twist, just yesterday, I found a link to an article. Apparently, it was a patient who had discovered that thalidomide could be useful for this and fed the information to the drug company, as a result of which, this product that had almost no market value started selling again. They quadrupled the price over the next few years. They have made another $300 million. I do not remember what the exact number is. They acknowledged her in their annual report but have not agreed to give her anything. So, she is now suing them for a portion of the extra earnings.

### Audience Member #7

My last point is that kids are better at searching for information online than adults. Back in 1992, I started the first pediatric network online for kids here. You had a number of times where kids taught parents or adults with cancer where to look for the information.

### Dave deBronkart

Terrific! That is great! That is great! See, we talk about the physicians who just do not want to use email, etc. Well, as time goes by, I know how that is going to unfold. Those people are retiring or dying and the others are coming up. So, you know, the iPod generation will take over.

### Audience Member #8 (David Wiljer)

Hi, David Wilder from Princess Margaret Hospital here in Toronto. Thank you very much for your talk, but also for some valuable tools. We are doing a lot of work in the field of trying to give patients access to their data. One of the things we often hear is “What are patients actually going to do with it?” So, the next time I get that question, I am just going to send them to your e-Patients.net website. So, thank you for that.

You hit on a very important point though, and I think we need to reflect on that: Giving data in this way—what you are advocating for—I think requires a fundamental shift around a lot of different things, but fundamentally around traditional definitions of privacy, security, confidentiality, and a very fundamental idea of what it means for the Hippocratic oath of “we shall not do harm.” For when we try to do this, we often hear clinicians say, “what we’re very concerned about what harm we might do from this perspective.” So, can you maybe give us a few thoughts on how we change this paradigm and how we are going to go about doing that? Thank you.

### Dave deBronkart

Deep question, and it is one where I am not an expert. The issue of privacy and the risk of people having their hands on their data, it kind of gets back to the Jefferson subject of when people are new to something, they may make mistakes, but there is a learning process, and you focus on getting to the goal. I know HIPAA is a vast subject, and I also know that HIPAA is a mess because there are things like this story: David Kibby, who is a well-known physician, came back from a conference this summer and reported that he had heard from an attorney that somehow that woman had somebody else’s data end up in her record, and the HIPAA goons at that hospital then locked her out of her own record because it contained data she was not supposed to see. Now, that is stupid.

At the same time, there was just a post last week on e-Patients.net by Susannah Fox about an article “Broken Promises of Privacy” from Paul Ohm, saying that the idea of “anonymized” data is actually a fallacy. Basically, smart hackers can de-anonymize data; the post starts by saying, “If you hate HIPAA, this is your lucky day,” because real new evidence came along. The other thing is, the part of HIPAA that is supposed to give us our data does not work out well, because it can take up to 2 months and there can be a ridiculous charge for it. What I have started doing is asking for copies of my own records myself and putting them in my own system that is outside HIPAA. Ironically, one of the breakdowns in HIPAA, from what I have heard, again, I am not an expert, is that the way the law was changed in 2002, you may have given permission or your hospital may have given permission to partners of theirs to look at your data and do things with it without you knowing it. Ironically, your data may be more secure outside of that system. Personally, I am taking matters into my own hand. I am going to take responsibility for my own medical record. I am not sure how it will all play out, but I think we need something different than the way it works today.

### Audience Member #9 (Peter)

Hi, my name is Peter. I too would like to thank you for the terrific presentation today. I just have two very quick questions:

First, has the integration between Beth Israel Deaconness’ Health, you know, patient portal and Google Health improved since it began with you?

Second, maybe if you could just speak to the value for you, personally, of Google Health as a place to store information beyond Beth Israel Deaconness’ circle of care?

### Dave deBronkart

So, the answer to the first one is yes and no. Once it became national news, they did stop transmitting billing codes. Interestingly, it turns out that a bunch of people complained about that because when their well-managed billing codes can be useful at least as a starting point. It turns out that Microsoft Healthvault takes a whole different approach to it. With Google Health, you punch the button and your data is there and that is that, for better or worse. With Healthvault, everything that you send in from Walgreens or your hospital or whatever goes into a holding bin, where you look at it and inspect it and then you say, “Okay, send it over.” Sounds to me like that is a much more sensible workflow. We do the same thing when we are putting a mailing list into our marketing system: We look at the stuff before we put it in because otherwise we end up spraying garbage on the walls, which, inside a computer, is messy.

Regarding the second question, the value for me, personally, of Google Health as a place to store information. If you go back and look at my April 1, 2009, blog post on e-Patients.net, what I see is, long story short, I want the power of innovation to be unleashed. However, innovative tools depend on data that they can work on. I mean, imagine, iPods would not have taken off much without digitized songs, and it is absolutely the same thing.

We are out of time, and I would again like to thank Gunther Eysenbach for inviting me to do this, and, as he said, as soon as that story hit the paper, he immediately grabbed me, and I am honored. Thanks very much to all of you.

## Appendix

Multimedia Appendix 1 Medicine 2.0’09 Opening Talk by Gunther Eysenbach (part 1).

Multimedia Appendix 2 “Gimme my damn data!” - e-Patient Dave’s keynote at Medicine 2.0 2009.

## Abbreviations

AMA: American Medical Association
ACOR: Association of Cancer Online Resources
HIPAA: Health Insurance Portability and Accountability Act
HIT: health information technology
IT: information technology
JMIR: Journal of Medical Internet Research
MIB: Medical Information Bank
